# 5-Ethyl-2-(4-fluoro­phen­yl)-4-phen­oxy-1*H*-pyrazol-3(2*H*)-one

**DOI:** 10.1107/S1600536811000754

**Published:** 2011-01-22

**Authors:** Tara Shahani, Hoong-Kun Fun, R. Venkat Ragavan, V. Vijayakumar, M. Venkatesh

**Affiliations:** a X-ray Crystallography Unit, School of Physics, Universiti Sains Malaysia, 11800 USM, Penang, Malaysia; b Organic Chemistry Division, School of Advanced Sciences, VIT University, Vellore 632 014, India

## Abstract

In the title compound, C_17_H_15_FN_2_O_2_, the essentially planar pyrazole ring [maximum deviation = 0.026 (1) Å] makes dihedral angles of 72.06 (7) and 33.05 (7)°, with the phenyl and fluoro­benzene rings, respectively. The dihedral angle between the two six-membered rings is 87.88 (7)°. In the crystal, inter­molecular N—H⋯O and C—H⋯F hydrogen bonds link the mol­ecules into layers lying parallel to the *bc* plane.

## Related literature

For pyrazole derivatives and their microbial activity, see: Ragavan *et al.* (2009 ▶, 2010 ▶). For the synthesis, see: Ragavan *et al.* (2009 ▶). For related structures, see: Shahani *et al.* (2009 ▶, 2010*a* ▶,*b* ▶). For hydrogen-bond motifs, see: Bernstein *et al.* (1995 ▶). For bond-length data, see: Allen *et al.* (1987 ▶). For the stability of the temperature controller used in the data collection, see: Cosier & Glazer (1986 ▶).
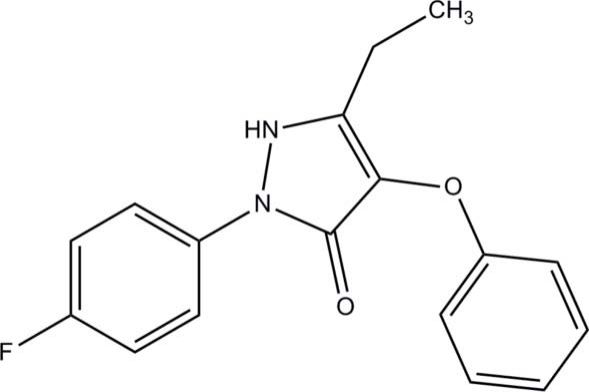


## Experimental

### 

#### Crystal data

C_17_H_15_FN_2_O_2_
*M* *_r_* = 298.31
Monoclinic, 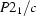

*a* = 15.332 (2) Å
*b* = 8.6833 (14) Å
*c* = 11.6066 (19) Å
β = 109.916 (3)°
*V* = 1452.8 (4) Å^3^
*Z* = 4
Mo *K*α radiation
μ = 0.10 mm^−1^
*T* = 100 K
0.55 × 0.14 × 0.08 mm

#### Data collection

Bruker APEXII DUO CCD diffractometer
Absorption correction: multi-scan (*SADABS*; Bruker, 2009 ▶) *T*_min_ = 0.947, *T*_max_ = 0.992
12927 measured reflections
4247 independent reflections
3207 reflections with *I* > 2σ(*I*)
*R*_int_ = 0.036

#### Refinement

*R*[*F* ^2^ > 2σ(*F* ^2^)] = 0.044
*wR*(*F* ^2^) = 0.119
*S* = 1.05
4247 reflections
204 parameters
H atoms treated by a mixture of independent and constrained refinement
Δρ_max_ = 0.34 e Å^−3^
Δρ_min_ = −0.24 e Å^−3^

### 

Data collection: *APEX2* (Bruker, 2009 ▶); cell refinement: *SAINT* (Bruker, 2009 ▶); data reduction: *SAINT*; program(s) used to solve structure: *SHELXTL* (Sheldrick, 2008 ▶); program(s) used to refine structure: *SHELXTL*; molecular graphics: *SHELXTL*; software used to prepare material for publication: *SHELXTL* and *PLATON* (Spek, 2009 ▶).

## Supplementary Material

Crystal structure: contains datablocks global, I. DOI: 10.1107/S1600536811000754/hb5788sup1.cif

Structure factors: contains datablocks I. DOI: 10.1107/S1600536811000754/hb5788Isup2.hkl

Additional supplementary materials:  crystallographic information; 3D view; checkCIF report

## Figures and Tables

**Table 1:** Hydrogen-bond geometry (Å, °)

*D*—H⋯*A*	*D*—H	H⋯*A*	*D*⋯*A*	*D*—H⋯*A*
N2—H1*N*2⋯O2^i^	0.899 (18)	1.803 (18)	2.6865 (14)	167.1 (16)
C11—H11*A*⋯F1^ii^	0.93	2.52	3.3441 (16)	147

Symmetry codes: (i) 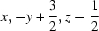; (ii) 
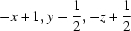
.

**Fig. 1.**
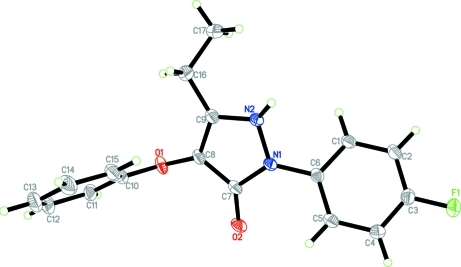
The molecular structure of (I), showing 50% probability displacement ellipsoids.

**Fig. 2.**
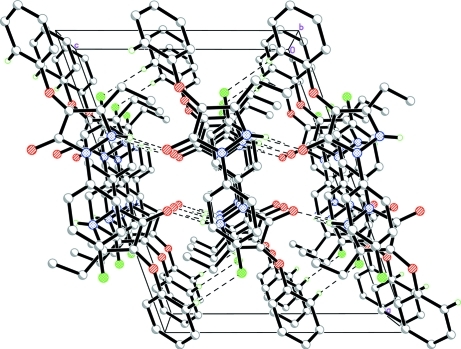
The crystal packing of (I), viewed along b axis. Intermolecular hydrogen bonds linked the molecules into two-dimensional arrays parallel to the bc plane. H atoms not involved in hydrogen bonds (dashed lines) have been omitted for clarity.

## supplementary crystallographic information

### Comment

Antibacterial and antifungal activities of the azoles are most widely studied and some of them are in clinical practice as anti-microbial agents. However, the azole-resistant strain had led to the development of new antimicrobial compounds. In particular pyrazole derivatives are extensively studied and used as antimicrobial agents. Pyrazole is an important class of heterocyclic compounds and many pyrazole derivatives are reported to have the broad spectrum of biological properties, such as anti-inflammatory, antifungal, herbicidal,anti-tumour, cytotoxic, molecular modelling, and antiviral activities. Pyrazole derivatives also act as antiangiogenic agents, A3 adenosine receptor antagonists, neuropeptide YY5 receptor antagonists, kinase inhibitor for treatment of type 2 diabetes, hyperlipidemia, obesity, and thrombopiotinmimetics. Recently urea derivatives of pyrazoles have been reported as potent inhibitors of p38 kinase. Since the high electronegativity of halogens (particularly chlorine and fluorine) in the aromatic part of the drug molecules play an important role in enhancing their biological activity, we are interested to have 4-fluoro or 4-chloro substitution in the aryls of 1,5-diaryl pyrazoles. As part of our on-going research aiming the synthesis of new antimicrobial compounds, we have reported the synthesis of novel pyrazole derivatives and their microbial activities (Ragavan *et al.*, 2009; 2010). The structure of the title compound, (I), is presented here.

In the title compound (Fig. 1), the molecule consists of two phenyl (C10—C15 and C1—C6) and one pyrazole (N1/N2/C7—C9) rings, all rings are essentially planar. The pyrazole ring (maximum deviation of 0.026 (1) Å at atom N1) makes dihedral angles of 72.06 (7) and 33.05 (7)°, with phenyl (C10—C15) and fluoro substituted phenyl (C1—C6) rings, respectively. The dihedral angle between the two six-membered rings,(C1—C6) and (C10—C15), is 87.88 (7)°. The bond lengths (Allen *et al.*, 1987) and angles are within normal ranges and are comparable to the closely related structures (Shahani *et al.*,2009; 2010*a*,b).

In the crystal (Fig. 2), intermolecular N2–H1N2···O2 and C11–H11A···F1 (Table 1) hydrogen bonds link the molecules into two-dimensional arrays parallel to the *bc* plane.

### Experimental

The compound was synthesized using the literature method (Ragavan *et al.*,2009) and recrystallized using an ethanol-chloroform 1:1 mixture to yield colourless needles of (I). Yield: 61%. *M.p.*: 441 K.

### Refinement

The hydrogen atom bound to the N2 atom was located in a difference map and allow to refine freely [N–H = 0.899 (18) Å]. All other H atoms were positioned geometrically [range of C–H = 0.93 to 0.97 Å] with *U*_iso_(H) = 1.2 or 1.5 *U*_eq_(C). A rotating-group model was applied for the methyl group

### Crystal data

**Table:**

C_17_H_15_FN_2_O_2_	*F*(000) = 624
*M**_r_* = 298.31	*D*_x_ = 1.364 Mg m^−^^3^
Monoclinic, *P*2_1_/*c*	Mo *K*α radiation, λ = 0.71073 Å
Hall symbol: -P 2ybc	Cell parameters from 2971 reflections
*a* = 15.332 (2) Å	θ = 3.0–33.0°
*b* = 8.6833 (14) Å	µ = 0.10 mm^−^^1^
*c* = 11.6066 (19) Å	*T* = 100 K
β = 109.916 (3)°	Needle, colourless
*V* = 1452.8 (4) Å^3^	0.55 × 0.14 × 0.08 mm
*Z* = 4	

### Data collection

**Table:**

Bruker APEXII DUO CCD diffractometer	4247 independent reflections
Radiation source: fine-focus sealed tube	3207 reflections with *I* > 2σ(*I*)
graphite	*R*_int_ = 0.036
φ and ω scans	θ_max_ = 30.0°, θ_min_ = 3.0°
Absorption correction: multi-scan (*SADABS*; Bruker, 2009)	*h* = −21→20
*T*_min_ = 0.947, *T*_max_ = 0.992	*k* = −12→9
12927 measured reflections	*l* = −16→16

### Refinement

**Table:**

Refinement on *F*^2^	Primary atom site location: structure-invariant direct methods
Least-squares matrix: full	Secondary atom site location: difference Fourier map
*R*[*F*^2^ > 2σ(*F*^2^)] = 0.044	Hydrogen site location: inferred from neighbouring sites
*wR*(*F*^2^) = 0.119	H atoms treated by a mixture of independent and constrained refinement
*S* = 1.05	*w* = 1/[σ^2^(*F*_o_^2^) + (0.047*P*)^2^ + 0.4776*P*] where *P* = (*F*_o_^2^ + 2*F*_c_^2^)/3
4247 reflections	(Δ/σ)_max_ < 0.001
204 parameters	Δρ_max_ = 0.34 e Å^−^^3^
0 restraints	Δρ_min_ = −0.24 e Å^−^^3^

### Special details

**Table:**

Experimental. The crystal was placed in the cold stream of an Oxford Cyrosystems Cobra open-flow nitrogen cryostat (Cosier & Glazer, 1986) operating at 100.0 (1) K.
Geometry. All e.s.d.'s (except the e.s.d. in the dihedral angle between two l.s. planes) are estimated using the full covariance matrix. The cell e.s.d.'s are taken into account individually in the estimation of e.s.d.'s in distances, angles and torsion angles; correlations between e.s.d.'s in cell parameters are only used when they are defined by crystal symmetry. An approximate (isotropic) treatment of cell e.s.d.'s is used for estimating e.s.d.'s involving l.s. planes.
Refinement. Refinement of *F*^2^ against ALL reflections. The weighted *R*-factor *wR* and goodness of fit *S* are based on *F*^2^, conventional *R*-factors *R* are based on *F*, with *F* set to zero for negative *F*^2^. The threshold expression of *F*^2^ > σ(*F*^2^) is used only for calculating *R*-factors(gt) *etc*. and is not relevant to the choice of reflections for refinement. *R*-factors based on *F*^2^ are statistically about twice as large as those based on *F*, and *R*- factors based on ALL data will be even larger.

### Fractional atomic coordinates and isotropic or equivalent isotropic displacement parameters (Å^2^)

**Table:**

	*x*	*y*	*z*	*U*_iso_*/*U*_eq_	
F1	0.77686 (5)	0.90535 (11)	0.09196 (7)	0.0332 (2)	
O1	0.22938 (6)	0.55499 (11)	0.07275 (8)	0.0226 (2)	
O2	0.40598 (6)	0.72722 (11)	0.20071 (7)	0.0243 (2)	
N1	0.41488 (7)	0.72695 (13)	0.00442 (8)	0.0187 (2)	
N2	0.36358 (7)	0.66422 (13)	−0.10812 (9)	0.0192 (2)	
C1	0.55898 (8)	0.70430 (15)	−0.03872 (10)	0.0191 (2)	
H1A	0.5320	0.6289	−0.0969	0.023*	
C2	0.64946 (9)	0.75124 (16)	−0.01831 (11)	0.0215 (3)	
H2A	0.6836	0.7098	−0.0634	0.026*	
C3	0.68736 (8)	0.86072 (16)	0.07039 (11)	0.0222 (3)	
C4	0.63952 (9)	0.92659 (15)	0.13937 (11)	0.0215 (3)	
H4A	0.6677	0.9992	0.1994	0.026*	
C5	0.54850 (8)	0.88198 (15)	0.11691 (10)	0.0200 (3)	
H5A	0.5142	0.9261	0.1607	0.024*	
C6	0.50869 (8)	0.77049 (14)	0.02820 (10)	0.0172 (2)	
C7	0.37115 (8)	0.69697 (15)	0.08861 (10)	0.0187 (2)	
C8	0.28708 (8)	0.62287 (15)	0.01905 (10)	0.0190 (2)	
C9	0.28418 (8)	0.60586 (15)	−0.09962 (10)	0.0190 (2)	
C10	0.14644 (8)	0.62813 (16)	0.06414 (11)	0.0204 (3)	
C11	0.10224 (9)	0.56911 (18)	0.14043 (11)	0.0254 (3)	
H11A	0.1284	0.4881	0.1933	0.030*	
C12	0.01814 (10)	0.6329 (2)	0.13677 (13)	0.0337 (4)	
H12A	−0.0120	0.5947	0.1880	0.040*	
C13	−0.02094 (10)	0.7526 (2)	0.05755 (14)	0.0378 (4)	
H13A	−0.0772	0.7950	0.0554	0.045*	
C14	0.02428 (10)	0.8092 (2)	−0.01883 (13)	0.0330 (3)	
H14A	−0.0023	0.8892	−0.0727	0.040*	
C15	0.10888 (9)	0.74771 (17)	−0.01581 (11)	0.0252 (3)	
H15A	0.1394	0.7863	−0.0665	0.030*	
C16	0.21135 (9)	0.53400 (17)	−0.20637 (11)	0.0246 (3)	
H16A	0.1673	0.6127	−0.2491	0.030*	
H16B	0.1781	0.4578	−0.1764	0.030*	
C17	0.25005 (10)	0.45693 (18)	−0.29685 (12)	0.0282 (3)	
H17A	0.2009	0.4051	−0.3590	0.042*	
H17B	0.2968	0.3836	−0.2542	0.042*	
H17C	0.2769	0.5336	−0.3342	0.042*	
H1N2	0.3689 (12)	0.706 (2)	−0.1764 (16)	0.035 (4)*	

### Atomic displacement parameters (Å^2^)

**Table:**

	*U*^11^	*U*^22^	*U*^33^	*U*^12^	*U*^13^	*U*^23^
F1	0.0199 (4)	0.0497 (6)	0.0311 (4)	−0.0092 (4)	0.0103 (3)	−0.0079 (4)
O1	0.0193 (4)	0.0301 (5)	0.0232 (4)	0.0039 (4)	0.0136 (3)	0.0076 (4)
O2	0.0251 (4)	0.0367 (6)	0.0138 (4)	0.0009 (4)	0.0100 (3)	−0.0019 (3)
N1	0.0179 (5)	0.0274 (6)	0.0124 (4)	−0.0018 (4)	0.0071 (4)	−0.0017 (4)
N2	0.0192 (5)	0.0275 (6)	0.0120 (4)	−0.0024 (4)	0.0067 (4)	−0.0009 (4)
C1	0.0208 (5)	0.0212 (6)	0.0166 (5)	0.0005 (5)	0.0080 (4)	−0.0013 (4)
C2	0.0211 (6)	0.0265 (7)	0.0200 (5)	0.0019 (5)	0.0109 (5)	−0.0001 (5)
C3	0.0179 (5)	0.0281 (7)	0.0202 (5)	−0.0016 (5)	0.0060 (4)	0.0021 (5)
C4	0.0220 (6)	0.0243 (6)	0.0174 (5)	−0.0009 (5)	0.0059 (4)	−0.0019 (4)
C5	0.0217 (6)	0.0237 (6)	0.0159 (5)	0.0031 (5)	0.0082 (4)	−0.0002 (4)
C6	0.0174 (5)	0.0209 (6)	0.0143 (5)	0.0013 (4)	0.0067 (4)	0.0027 (4)
C7	0.0203 (5)	0.0224 (6)	0.0160 (5)	0.0042 (5)	0.0097 (4)	0.0019 (4)
C8	0.0178 (5)	0.0244 (6)	0.0177 (5)	0.0016 (5)	0.0099 (4)	0.0023 (4)
C9	0.0181 (5)	0.0225 (6)	0.0177 (5)	0.0016 (5)	0.0078 (4)	0.0015 (4)
C10	0.0163 (5)	0.0283 (7)	0.0175 (5)	0.0002 (5)	0.0070 (4)	−0.0029 (4)
C11	0.0191 (6)	0.0394 (8)	0.0193 (6)	−0.0026 (5)	0.0087 (5)	0.0010 (5)
C12	0.0207 (6)	0.0588 (11)	0.0263 (6)	−0.0031 (7)	0.0142 (5)	−0.0045 (6)
C13	0.0218 (6)	0.0590 (11)	0.0344 (7)	0.0097 (7)	0.0119 (6)	−0.0071 (7)
C14	0.0295 (7)	0.0401 (9)	0.0288 (7)	0.0129 (6)	0.0090 (6)	0.0007 (6)
C15	0.0246 (6)	0.0314 (7)	0.0221 (6)	0.0039 (6)	0.0110 (5)	−0.0006 (5)
C16	0.0211 (6)	0.0321 (7)	0.0207 (6)	−0.0027 (5)	0.0071 (5)	−0.0019 (5)
C17	0.0301 (7)	0.0347 (8)	0.0194 (6)	−0.0042 (6)	0.0078 (5)	−0.0055 (5)

### Geometric parameters (Å, °)

**Table:**

F1—C3	1.3644 (14)	C8—C9	1.3708 (16)
O1—C8	1.3763 (15)	C9—C16	1.4928 (17)
O1—C10	1.3941 (15)	C10—C15	1.3809 (18)
O2—C7	1.2544 (14)	C10—C11	1.3837 (18)
N1—C7	1.3851 (15)	C11—C12	1.3907 (19)
N1—N2	1.3862 (13)	C11—H11A	0.9300
N1—C6	1.4202 (16)	C12—C13	1.382 (2)
N2—C9	1.3529 (16)	C12—H12A	0.9300
N2—H1N2	0.899 (18)	C13—C14	1.388 (2)
C1—C2	1.3866 (17)	C13—H13A	0.9300
C1—C6	1.3915 (17)	C14—C15	1.3922 (19)
C1—H1A	0.9300	C14—H14A	0.9300
C2—C3	1.3761 (18)	C15—H15A	0.9300
C2—H2A	0.9300	C16—C17	1.5255 (19)
C3—C4	1.3806 (18)	C16—H16A	0.9700
C4—C5	1.3848 (17)	C16—H16B	0.9700
C4—H4A	0.9300	C17—H17A	0.9600
C5—C6	1.3934 (17)	C17—H17B	0.9600
C5—H5A	0.9300	C17—H17C	0.9600
C7—C8	1.4206 (17)		
			
C8—O1—C10	118.93 (10)	N2—C9—C16	122.23 (11)
C7—N1—N2	109.59 (10)	C8—C9—C16	129.65 (12)
C7—N1—C6	127.91 (9)	C15—C10—C11	121.70 (12)
N2—N1—C6	119.98 (9)	C15—C10—O1	123.51 (11)
C9—N2—N1	108.32 (9)	C11—C10—O1	114.77 (11)
C9—N2—H1N2	124.6 (11)	C10—C11—C12	118.95 (13)
N1—N2—H1N2	118.8 (11)	C10—C11—H11A	120.5
C2—C1—C6	119.70 (11)	C12—C11—H11A	120.5
C2—C1—H1A	120.1	C13—C12—C11	120.48 (14)
C6—C1—H1A	120.1	C13—C12—H12A	119.8
C3—C2—C1	118.28 (12)	C11—C12—H12A	119.8
C3—C2—H2A	120.9	C12—C13—C14	119.58 (14)
C1—C2—H2A	120.9	C12—C13—H13A	120.2
F1—C3—C2	118.39 (12)	C14—C13—H13A	120.2
F1—C3—C4	118.40 (11)	C13—C14—C15	120.79 (14)
C2—C3—C4	123.20 (12)	C13—C14—H14A	119.6
C3—C4—C5	118.38 (12)	C15—C14—H14A	119.6
C3—C4—H4A	120.8	C10—C15—C14	118.50 (13)
C5—C4—H4A	120.8	C10—C15—H15A	120.8
C4—C5—C6	119.55 (11)	C14—C15—H15A	120.8
C4—C5—H5A	120.2	C9—C16—C17	113.48 (11)
C6—C5—H5A	120.2	C9—C16—H16A	108.9
C1—C6—C5	120.86 (11)	C17—C16—H16A	108.9
C1—C6—N1	119.90 (11)	C9—C16—H16B	108.9
C5—C6—N1	119.23 (11)	C17—C16—H16B	108.9
O2—C7—N1	123.71 (11)	H16A—C16—H16B	107.7
O2—C7—C8	131.88 (11)	C16—C17—H17A	109.5
N1—C7—C8	104.34 (10)	C16—C17—H17B	109.5
C9—C8—O1	127.09 (11)	H17A—C17—H17B	109.5
C9—C8—C7	109.42 (11)	C16—C17—H17C	109.5
O1—C8—C7	122.36 (10)	H17A—C17—H17C	109.5
N2—C9—C8	108.11 (10)	H17B—C17—H17C	109.5
			
C7—N1—N2—C9	4.94 (14)	O2—C7—C8—C9	−174.87 (14)
C6—N1—N2—C9	168.32 (11)	N1—C7—C8—C9	2.11 (14)
C6—C1—C2—C3	1.35 (18)	O2—C7—C8—O1	−6.2 (2)
C1—C2—C3—F1	179.00 (11)	N1—C7—C8—O1	170.74 (11)
C1—C2—C3—C4	−0.4 (2)	N1—N2—C9—C8	−3.48 (14)
F1—C3—C4—C5	179.60 (11)	N1—N2—C9—C16	177.94 (12)
C2—C3—C4—C5	−1.0 (2)	O1—C8—C9—N2	−167.13 (12)
C3—C4—C5—C6	1.43 (18)	C7—C8—C9—N2	0.83 (15)
C2—C1—C6—C5	−0.93 (18)	O1—C8—C9—C16	11.3 (2)
C2—C1—C6—N1	177.66 (11)	C7—C8—C9—C16	179.27 (13)
C4—C5—C6—C1	−0.49 (18)	C8—O1—C10—C15	13.96 (18)
C4—C5—C6—N1	−179.09 (11)	C8—O1—C10—C11	−167.62 (11)
C7—N1—C6—C1	137.38 (13)	C15—C10—C11—C12	−0.4 (2)
N2—N1—C6—C1	−22.65 (17)	O1—C10—C11—C12	−178.89 (12)
C7—N1—C6—C5	−44.01 (18)	C10—C11—C12—C13	0.4 (2)
N2—N1—C6—C5	155.96 (11)	C11—C12—C13—C14	0.1 (2)
N2—N1—C7—O2	173.06 (12)	C12—C13—C14—C15	−0.6 (2)
C6—N1—C7—O2	11.4 (2)	C11—C10—C15—C14	−0.1 (2)
N2—N1—C7—C8	−4.24 (13)	O1—C10—C15—C14	178.25 (12)
C6—N1—C7—C8	−165.94 (12)	C13—C14—C15—C10	0.6 (2)
C10—O1—C8—C9	−87.43 (16)	N2—C9—C16—C17	30.45 (18)
C10—O1—C8—C7	106.04 (13)	C8—C9—C16—C17	−147.80 (14)

### Hydrogen-bond geometry (Å, °)

**Table:**

*D*—H···*A*	*D*—H	H···*A*	*D*···*A*	*D*—H···*A*
N2—H1N2···O2^i^	0.899 (18)	1.803 (18)	2.6865 (14)	167.1 (16)
C11—H11A···F1^ii^	0.93	2.52	3.3441 (16)	147

Symmetry codes: (i) *x*, −*y*+3/2, *z*−1/2; (ii) −*x*+1, *y*−1/2, −*z*+1/2.
